# Opportunities and Challenges in North-South and South-South Global Health Collaborations During the COVID-19 Pandemic: The AFREhealth-CUGH Experience (as Reported at the CUGH 2021 Satellite Meeting)

**DOI:** 10.5334/aogh.3440

**Published:** 2021-09-09

**Authors:** Quentin Eichbaum, Nadia A. Sam-Agudu, Abigail Kazembe, Elsie Kiguli-Malwadde, Judy Khanyola, Judith N. Wasserheit, Peter H. Kilmarx, Jean B. Nachega

**Affiliations:** 1 Vanderbilt University Medical Center, Nashville, TN, USA; 2 International Research Center of Excellence, Institute of Human Virology Nigeria, Abuja, Nigeria; 3 Institute of Human Virology and Department of Pediatrics, University of Maryland School of Medicine, Baltimore, Maryland, USA; 4 Department of Pediatrics and Child Health, School of Medical Sciences, University of Cape Coast, Cape Coast, Ghana; 5 Kazembe – University of Malawi School of Nursing, MW; 6 African Centre for Global Health and Social Transformation, Kampala, Uganda; 7 University of Global Health Equity, Rwanda; 8 Department of Global Health, University of Washington Schools of Medicine & Public Health, Seattle, Washington; 9 Fogarty International Center, National Institutes of Health, Bethesda, Maryland, USA; 10 Department of Medicine and Center for Infectious Diseases, Stellenbosch University Faculty of Medicine and Health Sciences, Cape Town, South Africa; 11 Department of Epidemiology, Infectious Diseases and Microbiology, Center for Global Health, University of Pittsburgh, Pittsburgh, Pennsylvania, USA; 12 Department of Epidemiology, Johns Hopkins Bloomberg School of Public Health, Baltimore, Maryland, USA

## Abstract

Sustainable and equitable partnerships and collaborations between the Global North and Global South (as well as within the Global South) have been aspirations (if seldom achieved) of the “global health” endeavor over the past couple of decades. The COVID-19 pandemic led to global lockdowns that disrupted international travel and severely challenged these partnerships, providing a critical space for self-reflection on global health as a discipline. One major global north-south partnership is that between the African Forum for Research and Education in Health (AFREhealth) and the Consortium of Universities for Global Health (CUGH). This article reports on a recent Satellite meeting of the AFREhealth-CUGH Working Group (ACWG) at the CUGH 2021 virtual conference in March 2021 that provided insights on North-South and South-South global health partnerships, against the backdrop of the COVID-19 pandemic. The authors describe challenges and opportunities for research and education in these partnerships (as discussed at this ACWG Satellite meeting), and implications for the field of global health going forward as we emerge from the pandemic.

## Introduction

The COVID-19 pandemic led to global lockdowns that brought international travel to a sudden halt and disrupted many health-related collaborations between the Global North and Global South, and as well within the Global South. These disruptions created a space for questioning and self-reflection about the tenor and logistics of the global partnerships, and about the field of “global health” itself. Which aspects of partnerships in global health were indispensable, which could be adapted, substituted or abandoned? To what extent could information technology (IT) and internet connectivity substitute for in-person field collaborations to create new, and possibly more efficient and less costly, global networks of collaborators? To what extent has progress in global health research and education allowed the Global South to leapfrog ahead to create effective South-South collaborations among low- and middle income countries (LMICs) independent of the high income counties (HICs) of the Global North? [1] Is the Global South quite as dependent on the Global North as the latter has presumed, and what do these presumptions tell about the power dynamics of coloniality, the need to decolonize global health partnerships and, more broadly, about systemic racism and structural violence? The COVID-19 pandemic brought these questions into focus at a meeting in March this year between two major global health North-South partners – The Consortium of Universities for Global Health (CUGH) and the African Forum for Research and Education in Health (AFREhealth). In this article, we report on this Satellite meeting (of about a hundred participants) at the CUGH 2021 virtual conference that provided insights on North-South and South-South global health partnerships, against the backdrop of the COVID-19 pandemic.

### The AFREhealth Consortium

Established in 2016, the African Forum for Research and Education in Health (AFREhealth) is a pan-African organization that emerged from the Medical Education Partnership Initiative (MEPI) and Nursing Education Partnership Initiative (NEPI). MEPI and NEPI were funded by United States’ PEPFAR-and National Institutes of Health in 2010 and 2011, when awards were made to selected medical, nursing and health science schools in 13 African countries. AFREhealth, with its secretariat currently located in Accra, Ghana, seeks to work with Ministries of Health, training institutions and other stakeholders in Africa to improve the quality of healthcare across the continent through research, education, and capacity-building.

### The Consortium of Universities for Global Health (CUGH)

The Consortium of Universities for Global Health (CUGH) was established in 2008 and is a global network of almost 200 academic institutions worldwide, with administrative offices in Washington DC [2]. While most members are still US academic institutions, the organization continues expand its global membership in LMICs. The consortium supports its member organizations and partners to improve global health worldwide, through education, research, service, and advocacy. In conjunction with CUGH’s March 2021 virtual annual conference which focused on the theme “*Addressing Critical Gaps in Global Health and Development*,” 30 satellite meetings were convened. One of these was the AFREhealth-CUGH Working Group (ACWG) satellite at which participants from across Africa and from both high-income countries (HICs) and other low- and middle-income countries (LMICs) explored the opportunities and challenges of North-South and South-South global health collaborations [3,4]. The ACWG was established in 2017 (shortly after the founding of AFREhealth) as a supportive and collaborative endeavor between CUGH and AFREhealth. It is comprised of an Executive and three subcommittees – Education, Research and Student Exchanges – with co-chairs and members from each organization that seek to promote collaboration between the organizations in each of these three domains. The ACWG hosts its own Satellite meetings at both the CUGH and AFREhealth conferences each year, as well as separate committee meetings and webinars during the year.

Discussions focused on research, education, and student exchanges, especially in the wake of the COVID-19 pandemic. Dr. John Nkengasong, the Director of the Africa Centers for Disease Control and Prevention and the keynote speaker, addressed COVID-19 vaccine rollout in Africa and reflected on how CUGH and AFREhealth might collaborate in implementing and accelerating vaccine rollout as North-South global partners [3]. In this article, we expand on the AFREhealth-CUGH collaborative efforts and examine how such North-South and South-South collaborations can address key challenges and leverage opportunities for maximal impact.

### The COVID-19 Response in Africa: The Role of AFREhealth and CUGH

The keynote speaker Dr. Nkengasong highlighted major inequities in COVID-19 vaccine access and distribution in African countries, as reported elsewhere [5]. He outlined Africa CDC’s three-pronged approach to vaccine development and access: 1) accelerate Africa’s involvement in vaccine development, 2) secure continental access to a sufficient share of the global vaccine supply, and 3) remove barriers to vaccine scale-up across Africa [4]. An important component of the first approach he argued would be to develop a clinical trials network in Africa and to ensure that Africa participates in other COVID-19 vaccine trials to ensure vaccine safety and efficacy. The second approach would entail mobilizing capital from African member states as well as donors to secure sufficient and equitable doses of vaccine, and to scale up manufacturing capacity on the continent for long term local production of vaccines. The third approach would enable rapid vaccine approvals with ongoing safety monitoring, ensure functional supply chains and immunization activities, and enhance community engagement to maximize vaccine uptake.

He suggested that AFREhealth and CUGH have critical parts to play in prevailing upon governments and vaccine distributors to ensure adequate vaccine availability for African countries, in addition to educating the public about vaccines and countering vaccine hesitancy. Both organizations include healthcare leaders among their membership, who could advocate for increased vaccine availability and equitable distribution in African countries. We at AFREhealth and CUGH expect that such advocacy is likely to be increasingly impactful, given the current US administration’s re-engagement in global health, as evidenced by the US rejoining the World Health Organization (WHO).

## Rethinking Global Health Partnerships: Research and Education

The COVID-19 pandemic has given global health much pause for reflection. Over recent decades, the burgeoning field of global health has entailed frenzied travel between the Global North and Global South to implement collaborative projects in health research and teaching/education. Hundreds of collaborative endeavors were under threat because travel had been severely constrained or terminated. International research partners have had to rethink how to further their collaborations in this newly constrained environment [6]. How can research be conducted without field experiences, especially for international investigators, staff and students? How can teaching be done effectively without face-to-face interactions between teachers and learners? (The breakout session of the ACWG Education Committee discussed the benefits of virtual learning platforms that can reach more learners and is increasingly feasible with enhanced internet capacity on the continent).

As is now apparent, many aspects of research and teaching can be continued quite effectively via virtual platforms like Zoom and others. A successful example of the leverage of virtual video platforms for research collaborations is demonstrated by AFREhealth’s multi-country, South-South COVID-19 research project among pregnant women and children [7,8]. While deficiencies and concerns in education (especially clinical teaching and training) remain, several have been quite effectively addressed in some form through video-enhanced platforms like Lecturio, Osmosis, and Scholar-X [6]. Representatives from some of these companies (all of which have philanthropic engagements in Africa) gave demonstrations of their platforms in an education breakout session during the Satellite meeting [3]. How best to bring these virtual learning platforms to the service of interprofessional education (IPE) in Africa will be a major project for North-South collaboration of the ACWG in the near future.

A fundamental tenet of AFREhealth as an interprofessional organization is that the complex health problems facing the African continent can best be solved with all health professions working together as a team. IPE can enhance team-based care, while also enabling efficient use of training resources [9], leading to high impact in resource-constrained high disease burden settings [9]. An example of such an IPE model that evolved from face to face to e-learning platforms in the pandemic is the *Strengthening Inter-professional Education for HIV* (STRIPE-HIV) collaboration between AFREhealth and University of California San Francisco (UCSF) where a team of Nursing and Medical Educators developed a training package focused on clinical, public health, inter-professional Education (IPE), and quality improvement (QI) domains related to HIV service delivery [10].

The ACWG has worked in alignment with recommendations of the Lancet 2010 document *Health professionals for a New Century* to move from isolated to harmonized education and health systems and from stand-alone institutions to networks, alliances, and consortia [11,12]. But there have been challenges in working across continents and countries in different time zones; with asymmetries in North-South administrative and financial structures, differences in developmental infrastructures, and often with a dependence on foreign funding that continues to pose a threat to sustainability of projects.

### South-South and North-South Research Collaboration and Capacity Building

Regarding the COVID-19 pandemic, the relative paucity of published data from Africa on SARS-CoV-2 infection in the general population and from selected vulnerable groups (such as pregnant women and children from SSA) suggests an urgent need to combine case data across African countries in order to increase the sample size, statistical power and generalizability of results from the African continent. We need to conduct investigations that allow comparisons of predictors and outcomes across African countries and subregions. Such initiatives will also strengthen South-to-South multi-disciplinary and interprofessional research collaborations. AFREhealth’s COVID-19 Research Working Group has embarked upon such a collaboration across Western, Central, Eastern, and Southern Africa [7,8].

On the other side of the equation, the CUGH Research Committee’s goal is to facilitate avenues such as partnering with AFREhealth for expanding collaborative global health research and research training in priority areas, including pandemic research preparedness and response. For the 2021 CUGH virtual meeting, the CUGH Research and Education Committees, in collaboration with AFREhealth, piloted an Abstract Advising Program to connect early-career CUGH and AFREhealth members (advisees) with more experienced members (advisors). Once linked, they worked together in a relatively brief, focused interaction to improve the quality of the advisee’s abstract prior to submission for the CUGH conference. (A total of 50 members linked, 29 advisees with 21 advisors). An evaluation is underway to solicit suggestions for improvements and for future programs to facilitate similar, short-term “micro-mentoring” interactions between AFREhealth and CUGH members.

COVID-19 is already re-calibrating perspectives on levels of expertise in North-South relationships. The health systems in the COVID-19 response of several countries in the Global North underperformed in comparison to some countries in the Global South like Rwanda and Nigeria, especially in protecting vulnerable and marginalized populations. This is increasing momentum to democratize global health, with the recognition that humility and equity will be critical to learning and incorporating the lessons of COVID-19. In one such exchange of expertise, AFREhealth and the NIH Fogarty International Center co-sponsored a webinar on appropriate practices in contact tracing in Africa in October 2020, which was well attended by U.S. researchers and public health officials.

The AFREhealth and CUGH Research Committees place a high priority on building sustainable health research capacity in Africa, where the health burdens and threats are greater and research capacity is generally more limited than in higher-income countries. Human capacity is the critical pillar needed to establish robust, responsive research environments. We’re encouraged to see investigators trained in other research topics such as HIV and tuberculosis now emerge as leaders in Africa’s response to the COVID-19 pandemic. Other critical capacities include laboratory testing, data management and statistical analysis, clinical trial and community research site development, behavioral and social science, community engagement, ethical review boards and regulatory systems. As we have seen with COVID-19, these capacities can also be rapidly brought to bear to address new health threats. Notably, of the 30 countries taking part in the SOLIDARITY trial of COVID-19 treatment, 16 were LMICs, including two in Africa.^1^

### Coloniality and Power Asymmetries

The ghosts of colonialism and power dynamics of coloniality continue to haunt North-South partnerships despite vigorous recent debates about decolonizing global health [13,14,15]. Discussions about decolonizing global health are currently at the forefront of CUGH’s efforts to strengthen North-South partnerships in ways that are equitable and mutually beneficial.

The pandemic has illuminated specific power dynamics of coloniality and the inequities in current global health partnerships. While science has been an integral part of Western cultures, it is currently not as embedded in African cultures [16]. However, even after decades of North-South collaboration, funding and publication of research remain largely dominated by the Global North. Starting with MEPI and NEPI a decade ago and now with AFREhealth as the successor of those initiatives, empowerment of the Global South is edging forward at a steady, albeit slower than desired, pace.

Whereas high income countries of the Global North have, for decades, paid lip service to the need for bi-directional exchanges between countries of the Global North and South for effective collaboration, in practice exchanges have remained predominantly unidirectional from North (HICs) to South (LMICs). Indeed, the Global South may be relieved, to have fewer “global health experience” visits from the North that were often viewed by those from HICs as essential capacity building for LMICs. A smaller carbon footprint due to reduced travel is also welcomed by many in global health, and particularly by colleagues in Planetary Health. The northern colonialist metrics of global health – for example, how many countries we work in; how many global health grants we hold; how many students we “send” to the Global South – have also been called out by the pandemic for reflection and re-evaluation. What is global health about?

Ultimately, the COVID-19 pandemic may considerably transform the discipline of global health. There may be a healthy re-assessment and perhaps more equitable re-alignment of North-South partnerships that may benefit all partners. With this in mind, CUGH developed a survey that has been completed by about 250 programs globally to determine the impact of the COVID-19 pandemic on global health education and research. Results are currently being analyzed and should be illuminating in helping to identify lessons from the pandemic about global health teaching, research, funding, and other administrative components.

## Conclusions

The connection between AFREhealth and CUGH represents one of the largest collaborative efforts in health education and research between African countries of the Global South and countries of the Global North. While many asymmetries remain to be resolved in attaining equitable partnerships, the AFREhealth-CUGH partnership has made steady progress over the past five years and remains fully committed to strengthening health education and research on the African continent.

## Footnotes

**World Health Organization (WHO).** WHO Solidarity Trial Consortium. Repurposed Antiviral Drugs for Covid-19 – Interim WHO Solidarity Trial Results. N Engl J Med. 2020 Dec 2. doi: *10.1056/NEJMoa2023184*.
